# Exercise‐induced thoracic outlet syndrome and concomitant osteomyelitis in cervical rib with a possible familial origin: A case report

**DOI:** 10.1002/ccr3.5514

**Published:** 2022-04-22

**Authors:** Ammara Bint I Bilal, Mohammadshah Isam Gul, Fateen Ata, Renan E. Ibrahem, Muhammad I. Danjuma

**Affiliations:** ^1^ 36977 Department of Radiology Hamad Medical Corporation Doha Qatar; ^2^ 36977 Department of Internal Medicine Hamad Medical Corporation Doha Qatar; ^3^ College of Medicine Qatar University Doha Qatar

**Keywords:** accessory rib, cervical rib, familial, osteomyelitis

## Abstract

Cervical ribs are rare and usually asymptomatic. Occasionally, they can cause nerve impingements and compressive symptoms. In cervical ribs, osteomyelitis secondary to trauma is unheard of. We report such a case made more interesting by the familial presence of bilateral cervical ribs in two generations, indicating a familial origin.

## Graphical abstract

Cervical can present with thoracic outlet syndromes. Rarely, cervical ribs may present with osteomyelitis, which can be secondary to internal injury to the adjacent muscles. Additionally, cervical ribs may have a familial origin.

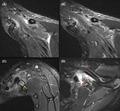

## 1 BACKGROUND

Cervical ribs, commonly addressed as accessory or neck ribs, are rare congenital supernumerary ribs arising from the seventh vertebra. This anatomic variant's prevalence is believed to be underreported as only symptomatic cases are brought to attention, whether unilateral or bilateral.^1^ A gender preference is observed, with the condition being twice as prevalent in women as men.^2^ Thoracic outlet syndrome (TOS) is caused by the compression of the subclavian artery/vein and/or the brachial plexus as they traverse through the thoracic outlet. TOS development is the leading clinical picture of discovery and morbidity associated with cervical ribs^1^; such ribs are large and frequently fused to the first rib. This placement also predisposes to aneurysm formation or thrombosis..^3^

Cervical ribs have been associated with mutations in the HOX genes responsible for constructing axial skeleton patterns.^4^ Owing to their rarity, it has been challenging to establish a familial pattern. Taku Suzuki et al. have previously presented clinical images of bilateral cervical ribs in a mother and daughter, hinting toward genetic predisposition, although the available data in the literature are trivial.^5^ In the early 20th century, a family was reported in which seven out of 11 members had cervical ribs, parents having unilateral, and five children with bilateral cervical ribs.^4^ Osteomyelitis of ribs (ORs) is rare and is usually associated with tuberculosis wherever seen.^6^ Rare cases have been reported of nontuberculous OR, but no current literature exists regarding cervical rib osteomyelitis. We present an interesting case of bilateral cervical ribs in a patient with fibromyalgia in which TOS was triggered by trauma, further complicated by osteomyelitis, and thorough history revealed a familial pattern of cervical ribs.

## 2 CASE PRESENTATION

A 26‐year‐old Pakistani woman presented to the emergency department with a 5‐day history of gradually increasing left shoulder pain for 1 week. She also had two episodes of fever at home, for which she took paracetamol. The patient was a known case of fibromyalgia, which was diagnosed in 2018. Her disease was stable on amitriptyline and gabapentin with physiotherapy. She also had postural tachycardia syndrome, for which she was taking bisoprolol 2.5 mg daily.

She was discharged a week ago from the rehabilitation institute for physiotherapy, where she stayed for a few days as a part of her fibromyalgia management. During her stay, the patient developed left shoulder pain as a result of intensive physiotherapy. She was diagnosed with having an internal injury to muscles adjacent to the cervical rib secondary to hyperextension during physical therapy. The pain was managed with tramadol oral 50 mg thrice daily as needed (PRN) for 2 days and celecoxib 200 mg twice daily for 5 days. She was discharged from the rehabilitation center on celecoxib.

The intensity of the pain at discharge from physical therapy rehabilitation was 3/10, which gradually increased to 8/10. She described it as an electric shock‐like sensation radiating from her left shoulder to the left hand. The patient also has an on and off burning sensation in the upper left arm. There was minimal movement in the left arm due to pain, which was not responding well to the analgesia. She denied cough, running nose, headache, chest pain, palpitation, abdominal pain, diarrhea, constipation, dysuria, urgency, or increase in frequency. Upon physical examination, the patient was afebrile (37.2^o^ C), with a heart rate of 96 beats/min, respiratory rate of 18/min, and blood pressure of 109/73 mmHg (which was normal for her). She was maintaining normal saturation.

As she had two fever episodes at home, infection was thought of as a possibility, and an infectious disease workup was sent. Results showed leukocytosis of 22.1 × 10^3^/ul with an absolute neutrophil count of 18.2x10^3^/ul, normocytic anemia with hemoglobin of 11.6 gm/dl (on discharge it was 13.2gm/dL), and elevated platelets of 672 × 10^3^/ul (on discharge: 255 × 10^3^ul). She had a raised C‐reactive protein (CRP) level (118 mg/L) with a normal procalcitonin and lactic acid. Her kidney functions and electrolytes were within the normal range, and she had mildly elevated liver functions (alanine aminotransferase: 139 U/L, aspartate aminotransferase: 38 U/L, and alkaline phosphatase 286 U/L). Blood and urine bacterial cultures were sent, and the patient was started on ceftriaxone intravenously (IV) 2 gm daily, tramadol 50 mg oral PRN q8 h, and regular paracetamol 1 gram oral q6 h.

**FIGURE 1:**
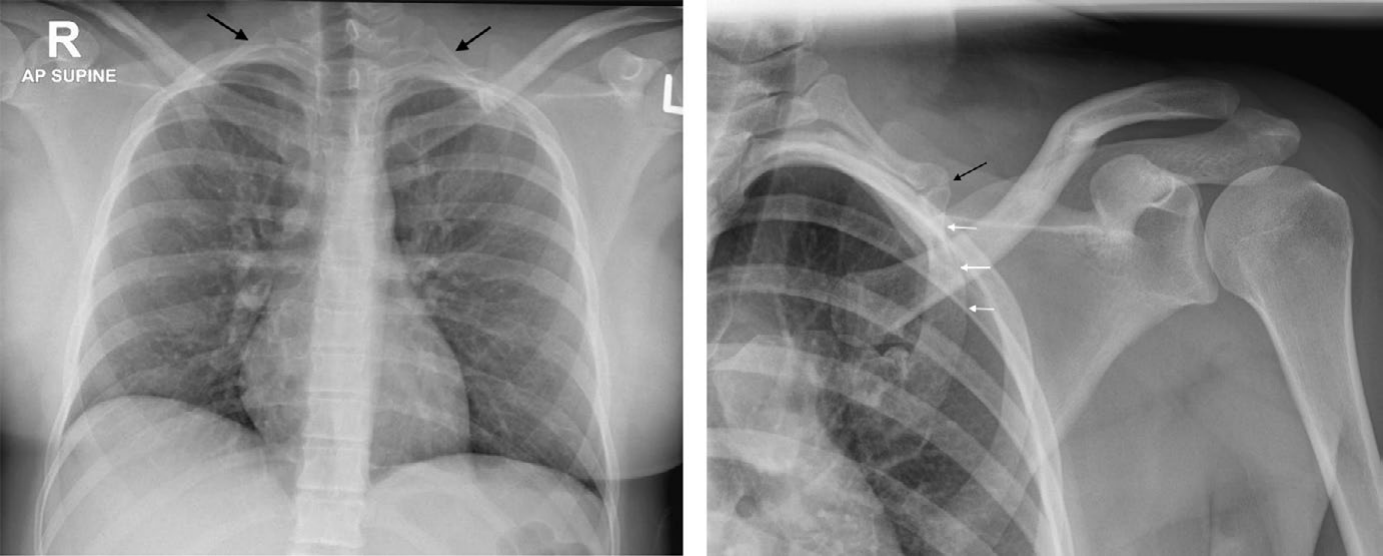
Left (A) AP chest radiograph shows bilateral cervical ribs. Right (B) AP radiograph of the left shoulder shows pseudoarthrosis (black arrow) of the cervical rib with the deformed first rib (white arrows)

Before admission, she had visited a private clinic where an ultrasound of the left shoulder was performed, which was reported as marked heterogenicity of the scalene muscle of left with surrounding soft tissue edema and irregular collection (10 ml) toward the root of the neck. X‐ray of the chest (CXR) and left shoulder showed no fracture; however, it revealed bilateral complete cervical ribs (Figure 1A,B).

**FIGURE 2:**
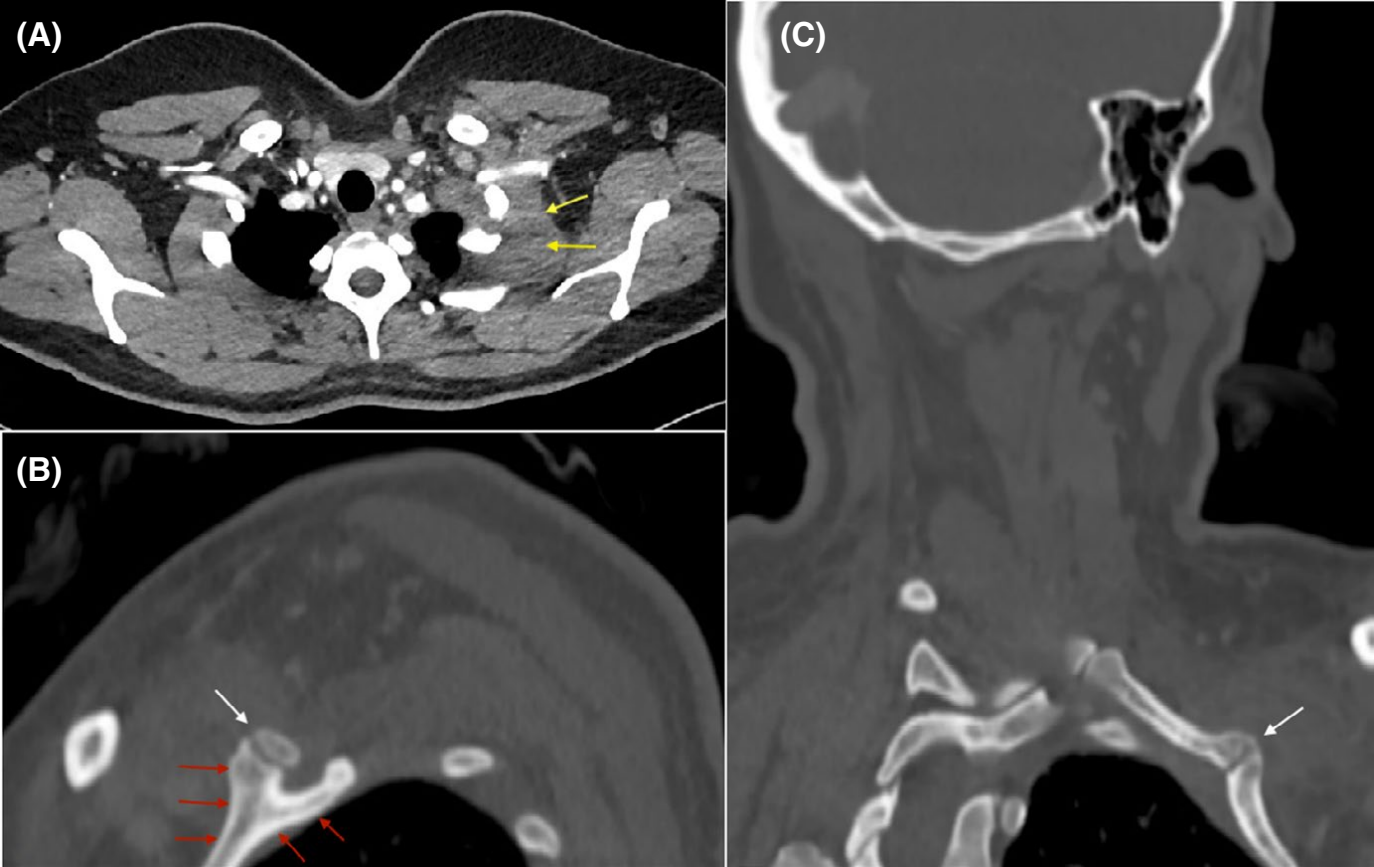
CT chest. (A) The axial CT image at the apices’ level demonstrates thickening of the left scalene muscle with associated soft tissue thickening/hematoma around the left cervical rib's pseudoarthrosis with the first rib (yellow arrows). (B) Sagittal and (C) oblique coronal CT images of the left side of the chest show deformity of the left first rib with bifid appearance anteriorly (red arrows) and associated pseudoarthrosis with the anterior end of the cervical rib (white arrow)

Given the findings of the ultrasound with a suspicion of possible infective collection (due to the fever episodes) or hematoma (as she had a drop in hemoglobin level), a computerized tomography (CT) scan of the neck and thorax was performed. It revealed a bulky appearing left scalene muscle (6 cm × 3 cm in maximum) with necrotic component or hematoma measuring 21 mm × 18 mm, associated with fat stranding and left supraclavicular lymph nodes. It also detailed the previously seen cervical ribs, with the left cervical rib deformed anteriorly and fused with the left first rib (pseudoarthrosis) (Figure 2).

Blood and urine cultures came positive for Staphylococcus aureus (S. aureus) by day 3. Her antibiotics were changed to cefazolin intravenous (IV) q8 h and one shot of vancomycin 1.5 gm IV (while awaiting S. aureus sensitivity, which eventually turned to be methicillin‐sensitive).

**FIGURE 3:**
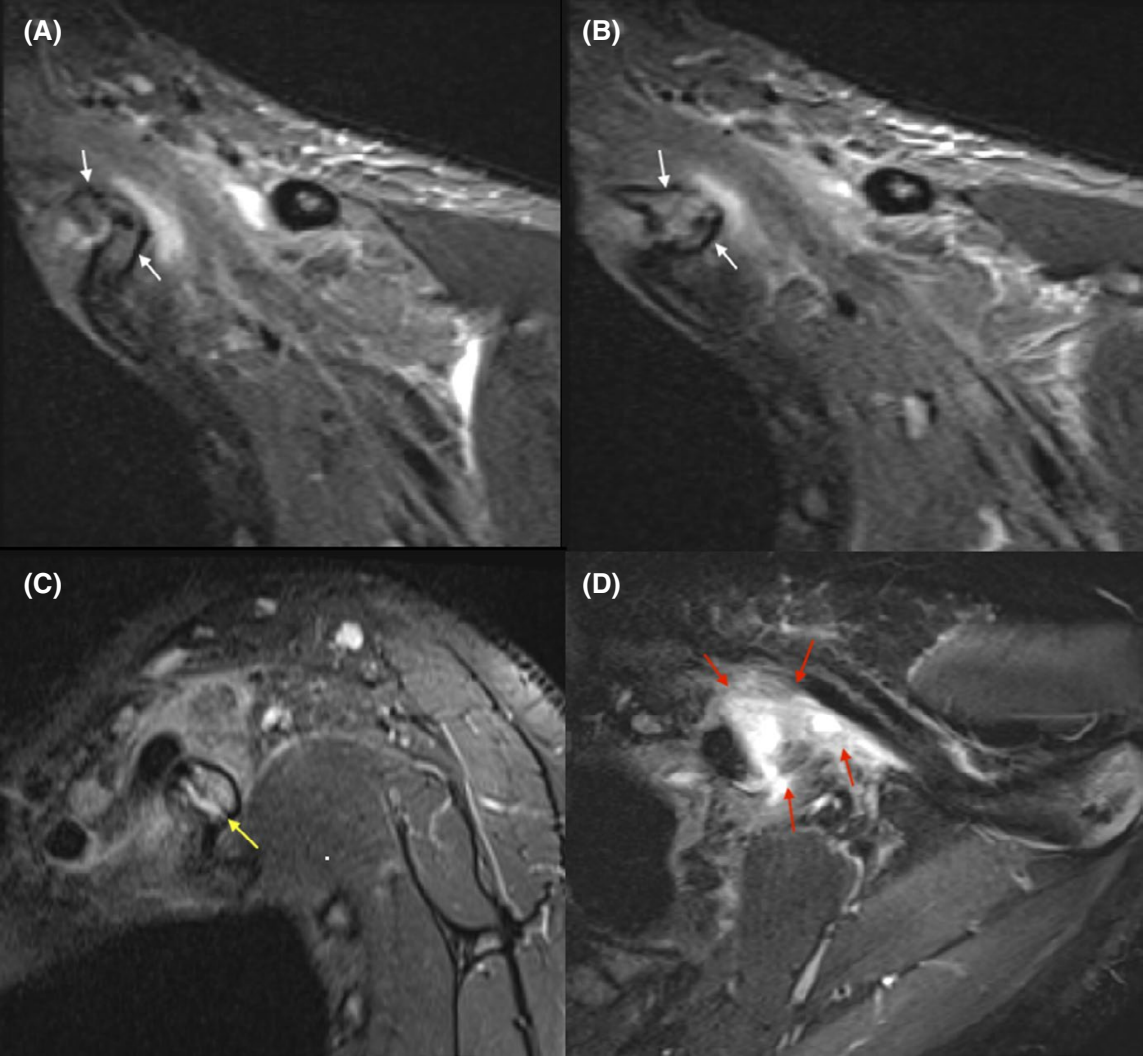
MRI of the left shoulder. (A and B) Coronal STIR images show marrow edema at both ends of the pseudoarthrosis involving the cervical and adjacent first ribs (white arrows). (C) Sagittal and (D) axial STIR images show a trace of fluid in the pseudoarthrosis region (yellow arrow) with surrounding soft tissue edema associated with small hematoma/fluid collection (red arrows)

Magnetic resonance imaging (MRI) of the left shoulder was performed to evaluate better the necrotic component seen previously in the CT scan and to rule out septic arthritis as a possible diagnosis. The shoulder joint appeared normal. The collection appeared to be related to the left cervical rib's pseudoarthrosis with the first deformed rib. It also showed focal low‐grade marrow edema involving the cervical rib. The findings were suspicious of osteomyelitis of the left cervical rib's distal end (Figure 3).

Thoracic surgery was consulted for the possibility of drainage; however, conservative treatment was recommended with an outpatient follow‐up.

A transthoracic echocardiogram was performed to rule out infective endocarditis; it showed no vegetation. Repeated blood cultures were negative by day 6. She completed 1 week of cefazolin IV q8 h. Her pain improved, and she was discharged on analgesics (paracetamol/orphenadrine 1000 mg q8 h, diclofenac sodium cream q12 h, and naproxen 250 mg q12 h PRN) for five more days and cephalexin 1 gm q8 h for another 5 weeks to complete the total duration of 6 weeks for the osteomyelitis.

## 3 DISCUSSION

Osteomyelitis is a medical emergency characterized by infection of the bones.^7^ The most commonly involved bones are long bones of arms and legs in children, whereas, in adults, the most commonly involved bones are pelvic bones, spine, and lower limbs.^8^ It may be caused by a multitude of microorganisms, including bacteria, viruses, parasites, and fungi. However, the most common association is with pyogenic bacteria and mycobacteria. Among the cases of pyogenic osteomyelitis, S. aureus is found in 80% to 90% of cases, whereas Staphylococcus epidermidis (which is the most prevalent skin flora) primarily tends to infect medical devices, including implants and catheters.^9^

Rib involvement for osteomyelitis is rare,^10^ with osteomyelitis of the cervical ribs in an adult not reported before, to the best of our knowledge. It is unknown whether an accessory rib inherently has increased, decreased, or similar predisposition to developing an infection of the bone and whether any specific bacteria are more likely to infect such anatomic variants. In our case, the patient developed S. aureus osteomyelitis. This area of cervical rib complications needs further exploration.

Cervical ribs narrow the boundaries of the interscalene triangle forming the inferior border before the first rib.^11^ This predisposes to the development of TOS, which can be neurogenic or vascular. This compression neuropathy is one of the most challenging neuropathies for neurosurgeons as no definitive guidelines exist as to when should the supernumerary rib be surgically excised. A variable clinical presentation arises with complete vs. incomplete cervical ribs as vascular symptoms are almost exclusive to complete cervical ribs.^1^

Without any acute neurological or vascular manifestation in symptomatic patients, treatment depends on the corresponding manifestations. All cases are treated conservatively by pain killers such as nonsteroidal anti‐inflammatory drugs (NSAIDs) and muscle relaxants and emphasizing lifestyle changes. Physiotherapy is encouraged to strengthen the muscles around the shoulder girdle. Surgery is considered if all conservative measures have failed, and it is the treating physician's clinical decision.^4^

Our patient developed radiating neuropathic pain following rehabilitation exercise for fibromyalgia, indicating a possible trigger of TOS. The question arises if such patients should be recommended to exercise with caution or if they should avoid certain movements that can narrow the interscalene triangle further? Again this area is unexplored yet contributes significantly to avoidable morbidity in patients with cervical ribs.

Although not established, the familial origin of cervical ribs has been proposed as a possibility. A meta‐analysis has demonstrated variability in the prevalence of cervical ribs with respect to geographical and ethnic diversities, supporting a crucial role of genetics.^1^ Furthermore, clinically, the presence of cervical ribs in families has long been accepted; however, the inheritance patterns remain unknown. In current literature, a study has suggested autosomal‐dominant inheritance as they found nine members spanning three generations of a South African family.^12^ In our case, the patient's brother and mother both have cervical ribs, whereas further family history is undocumented.

## 4 CONCLUSIONS

Understanding the predispositions and consequences of cervical ribs is vital to anticipate and effectively manage the complications associated with this genetic anomaly. Familial origin has been suggested multiple times, but no convincing evidence exists as it is still an underexplored area in medicine. Trauma, overuse, and poor posture are the leading causes that predispose individuals to symptoms. More extensive studies focusing on avoidable triggers are needed since the development of TOS confers a substantial decrease in quality of life. The pathophysiology behind cervical rib osteomyelitis remains unexplored, but in our case, since it was S. aureus induced, we can conclude that it possibly follows the same trends as any other osteomyelitis. Additionally, internal injury to adjacent muscles or ligaments by the cervical rib can lead to osteomyelitis, and hence physical therapy should be provided with excessive care in such patients. However, further research is required to make evidence‐based conclusions regarding the following:

- Is there a familial origin of cervical ribs? If so, what is the inheritance pattern?
- What are the avoidable triggers, for example, exercises that may narrow the scalene triangle further to induce TOS in patients with asymptomatic cervical ribs?
- Is osteomyelitis in cervical ribs any different from osteomyelitis in other ribs?

## CONFLICT OF INTEREST

The authors declare that they have no competing interests.

## AUTHOR CONTRIBUTIONS

(AB and MG share cofirst authorship due to equal contribution). AB contributed to literature review, manuscript writing, and radiological image selection. MG contributed to literature review, manuscript writing, and obtained written consent. RA contributed to radiology workup of the patient, finalization of the images, and figure legends. FA contributed to literature review, critical review, and revisions in the manuscript. MD contributed to supervision, literature review, critical review, and revisions in the manuscript. All authors read and approved the final version of this manuscript.

## ETHICAL APPROVAL

Ethical approval for this study was obtained from Medical Research Center (MRC) Qatar (MRC‐01–20–577).

## CONSENT

Written consent was taken from the patient for publication of data and accompanying images.

## DECLARATION

This manuscript is the original work and has not been submitted or is not under consideration for publication elsewhere. All the authors have reviewed the manuscript and approved it before submission. None of the authors have any conflicts of interest in publishing this work.

## Data Availability

​Available upon reasonable request from the corresponding author.
